# Identification of epistatic interactions between the human RNA demethylases FTO and ALKBH5 with gene set enrichment analysis informed by differential methylation

**DOI:** 10.1186/s12919-018-0122-0

**Published:** 2018-09-17

**Authors:** Elizabeth R. Piette, Jason H. Moore

**Affiliations:** Institute for Biomedical Informatics, Richards Medical Research Laboratories, Perelman School of Medicine, University of Pennsylvania, 3700 Hamilton Walk, Philadelphia, PA 19104 USA

## Abstract

The Genetic Analysis Workshop (GAW) presents an opportunity to collaboratively evaluate methodology relevant to current issues in genetic epidemiology. The GAW20 data combine real clinical trial data with fictitious epigenetic drug response endpoints. Considering the evidence suggesting that networks of interactions between many genes underlie complex phenotypes, we utilize differential methylation status to identify a relevant gene set for enrichment analysis and use this to infer potential biological function underlying drug response. We highlight the pertinence of considering the potential for widespread epistatic interactions in the absence of main effects, and present evidence of epistasis between single-nucleotide polymorphisms (SNPs) on the two RNA demethylases *FTO* and *ALKBH5*.

## Background

The GAW is a forum for investigators to develop and critique new analytical methods for complex traits on a shared data set. The GAW20 data provide simulated replications based on the Genetics of Lipid-Lowering Drugs and Diet Network (GOLDN) clinical trial, had participants been subject to treatment with a fictitious drug with a pharmacoepigenetic effect on triglyceride response [1]. These data present a unique analysis opportunity as all phenotypes, subject covariates, genotypes, pre-treatment methylation levels, etc. are real data from the trial, but are accompanied by simulated post-treatment methylation and triglyceride levels. GAW participants choose to analyze the data with or without knowing the simulation methods; we chose to perform the simulated data analysis prior to attending GAW, without knowledge of the simulation methods. Analysis of the real data was performed following GAW attendance, as it was revealed that the data simulation methods did not consider interactions, and therefore analysis of interactions in the simulated data was not appropriate.

Despite evidence for multi-locus underpinnings of phenotype-genotype association, the multiple-testing burden associated with fitting interaction models is stringent due to the high dimensionality of genomic data [2]. Strategies for better detecting these interactions can aim to avoid exhaustively testing each potential interaction via data reduction methods, integrating expert knowledge, and/or consolidating multiple sources of evidence to narrow the search space [3–5].

In this study, we hypothesize that CpG sites that are differentially methylated with respect to treatment are associated with the pharmacoepigenetic mechanism of the fictitious drug. Considering the evidence for multi-locus models of complex disease etiology, we hypothesize that the drug response is better evaluated by gene set enrichment analyses than by single locus models. By integrating results from gene ontology, drug-disease association, and microRNA (miRNA) target analyses we find evidence implicating the relevance of adenosine and miRNAs with known epigenetic regulation and roles in lipid metabolism. From this, we infer the potential importance of the N^6^-methyladenosine modification in the pharmacoepigenetic response on triglycerides, and consider how miRNA adenosine methylation rather than CpG methylation may impact the phenotype. Lacking direct data for miRNA adenosine methylation, we perform a targeted epistasis search between loci on the two RNA demethylases *FTO* and *ALKBH5*, and find evidence for statistical epistasis between one variant within each respective gene. Repeating the analysis with the real data revealed four significant interactions between variants across these genes. Overall, we present an example workflow in which integration of multiple sources of information can help uncover biological meaning in the absence of significant main effects.

## Methods

### Data set

The GOLDN data and companion simulations for GAW20 are previously described [1, 6]. Relevant to this analysis, subject data includes fasting lipid profiles prior to and post-treatment, methylation at more than 450,000 CpG sites prior to and post-treatment, GWAS of more than 700,000 autosomal SNPs, and covariates including age, center, metabolic syndrome-related traits, and smoking status. This analysis is of the pre-defined single representative replicate (*n* = 680) of the post-treatment methylation and triglyceride levels.

### Phenotype definition

We define the phenotype of interest as the log ratio of the average post-treatment triglyceride level to the average pre-treatment triglyceride level. Due to the high correlations between triglyceride levels at pre-treatment time points 1 and 2 and post-treatment time points 3 and 4 (0.90 and 0.91, respectively), and presence of a value for at least one of time point 1 or 2 and 3 or 4 for each individual, we singly impute missing values via linear regression.

### CpG site filtering

Significantly differentially methylated CpG sites are identified via paired *t-*tests for pre- and post-treatment methylation levels (α = 0.05 for 463,995 hypotheses yields Bonferroni cutoff of 1.08 × 10^− 7^).

### Modeling the relationship between phenotype and CpG site methylation

Linear models are fit to test the relationships between the phenotype and methylation status of the significant CpG sites identified above, characterized as a single predictor: the log ratio of post-treatment to pre-treatment methylation (α = 0.05 for 212,018 hypotheses yields Bonferroni cutoff of 2.36 × 10^− 6^).

### Gene set enrichment analyses

All CpG sites that pass the initial *t*-test filter and have a *p*-value ≤0.05 for the phenotype ~ methylation predictor model are used to curate a list of corresponding genes with evidence for both differential methylation and association with the phenotype. This gene list is used for gene ontology, drug association, and miRNA target enrichment analyses using the WEB-based GEneSeTAnaLysis Toolkit (WebGestalt, http://www.webgestalt.org/) [7].

### Targeted epistasis search

We investigate potential epistasis between the two RNA demethylases *FTO* and *ALKBH5* by calculating *p*-values for the likelihood ratio tests, comparing the linear models containing each *FTO*-*ALKBH5* SNP-SNP pair, with and without their interaction term (α = 0.05 for 340 hypotheses yields Bonferroni cutoff of 0.00015 for the simulated data; 255 hypotheses yields a cutoff of 0.000196 for the real data).

## Results

### CpG site filtering

We tested 463,995 CpG sites for differential methylation prior to versus post-treatment; 212,018 CpG sites passed the Bonferroni threshold of 1.08 × 10^− 7^.

### Modeling the relationship between phenotype and CpG site methylation

**Fig. 1:**
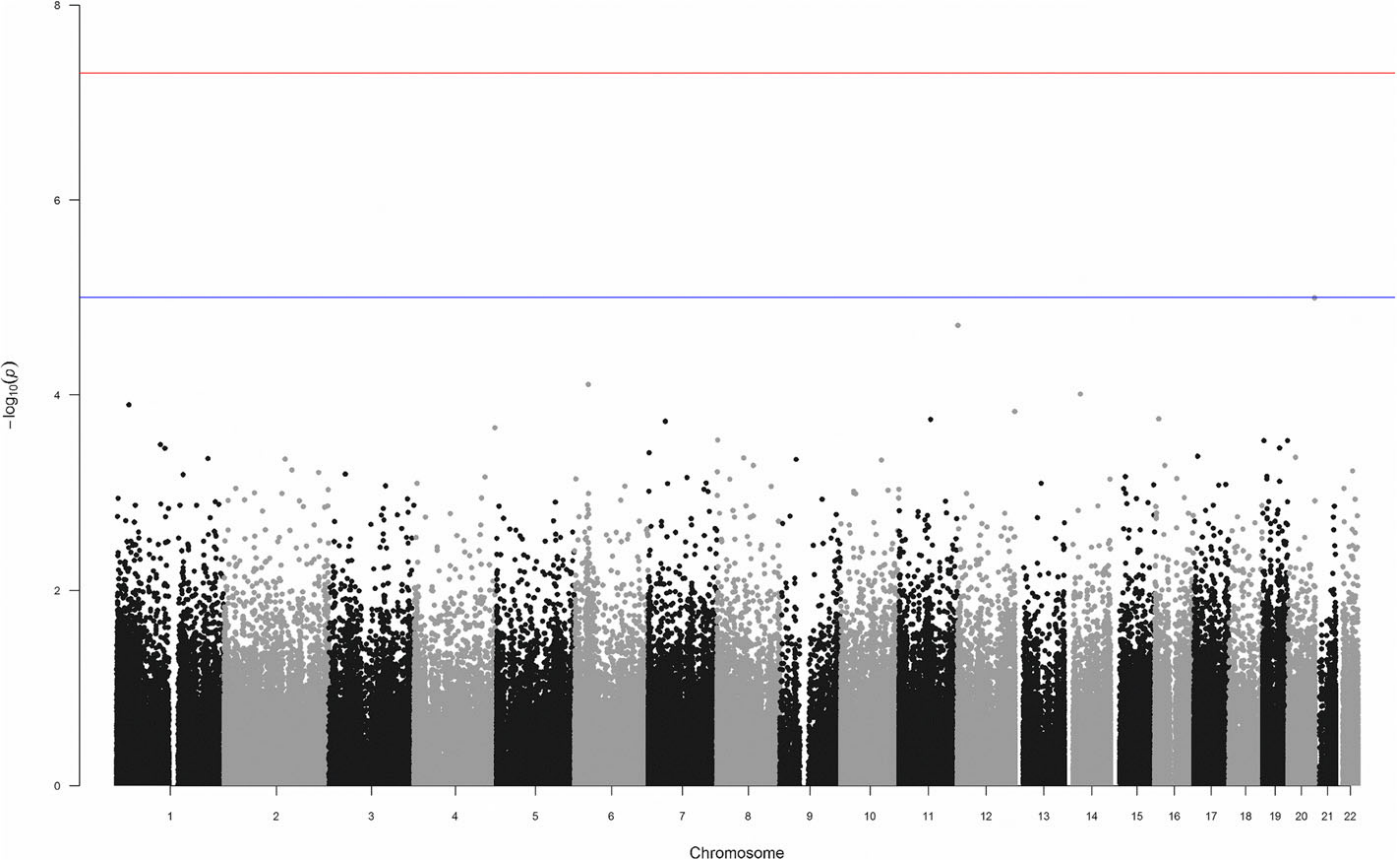
Manhattan plot of triglyceride phenotype ~ CpG site methylation log ratio

None of the 212,018 CpG sites that are significantly differentially methylated reached genome-wide significance for association with the phenotype (Fig. 1).

### Gene set enrichment analyses

Our gene set is constructed from all CpG sites with *p*-values ≤0.05 for the models above for which corresponding gene annotations are available (5413 of the 212,018 differentially methylated sites). Some CpGs have more than one corresponding gene listed, and many genes have multiple CpGs, for a total gene list length of 4443. The top result from the drug association analysis is adenosine (number of reference genes in the category = 477; number of genes in the gene set and also in the category = 126; expected number in the category = 49.14; ratio of enrichment = 2.56; raw *p*-value from hypergeometric test = 1.31 × 10^− 23^; *p*-value adjusted by multiple test adjustment = 8 × 10^− 21^). The top result from the miRNA target analysis is miR-124a (number of reference genes in the category = 542; number of genes in the gene set and also in the category = 175; expected number in the category = 55.84; ratio of enrichment = 3.13; raw *p*-value from hypergeometric test = 7.82 × 10^− 45^; *p*-value adjusted by multiple test adjustment = 1.70 × 10^− 42^).

### Targeted epistasis search

The GAW20 GWAS data with the simulated phenotype include complete observations for 68 SNPs on *FTO* and 5 SNPs on *ALKBH5* for the 680 subjects. We only test for epistatic interactions between SNPs across the two genes (and do not test for interactions between SNPs on the same gene), for a total of 340 tested interactions and therefore a Bonferroni threshold of 0.00015. One pair of SNPs, *rs2192872* from *FTO* and *rs8068517* from *ALKBH5*, have a significant *p*-value for the likelihood ratio test comparing the models with and without the interaction (*p* = 2.01 × 10^− 5^).

**Fig. 2:**
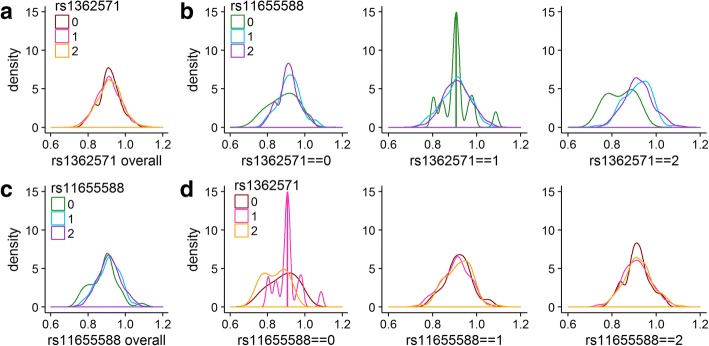
Phenotype distributions by genotype **a**. Phenotype distributions for individuals with 0, 1, or 2 copies of the minor allele for *rs1362571*. **b**. Phenotype distributions for individuals with 0, 1, or 2 copies of the minor allele for *rs11655588* among those with 0 copies of the minor allele for *rs1362571* (left); 1 copy (center); 2 copies (right). **c**. Phenotype distributions for individuals with 0, 1, or 2 copies of the minor allele for *rs11655588*. **d**. Phenotype distributions for individuals with 0, 1, or 2 copies of the minor allele for *rs1362571* among those with 0 copies of the minor allele for *rs11655588* (left); 1 copy (center); 2 copies (right)

The analysis of the real phenotype and GWAS data was performed in the same manner for 778 subjects for 51 SNPs on *FTO* and 5 SNPs on *ALKBH5* (Bonferroni threshold of 0.000196). Four pairs of SNPs have significant *p* values for the likelihood ratio test comparing the models with and without the interaction (Table 1). Figure 2 visually summarizes the distribution of phenotype by genotype for the two SNPs involved in the most significant identified interaction.

**Table 1:** Summary of significant interactions, Variant annotations are from Ensembl [29]. Base model covariate selection is based on significance at the 0.05 level and includes average pre-treatment triglyceride level, age, center, current smoker status, and sex

SNP	Alleles	MAF	Location	Gene	Consequence type	LRT *p*-value
*rs1362571*	G/T	0.34 (G)	16:53877858	*FTO*	Intron variant	2.76 × 10^− 6^
*rs11655588*	A/G	0.18 (G)	17:18204137	*ALKBH5*	Intron variant	
*rs10521304*	T/C	0.41 (C)	16:53874745	*FTO*	Intron variant	8.80 × 10^− 6^
*rs11655588*	A/G	0.18 (G)	17:18204137	*ALKBH5*	Intron variant	
*rs1421090*	A/G	0.29 (G)	16:53816258	*FTO*	Intron variant	0.000158
*rs8071834*	T/C	0.45 (C)	17:18196677	*ALKBH5*	Intron variant	
*rs17820875*	A/G	0.12 (G)	16:53892878	*FTO*	Intron variant	0.000177
*rs8068517*	G/A	0.24 (G)	17:18192664	*ALKBH5*	Intron variant	

*Abbreviations: LRT* likelihood ratio test, *MAF* minor allele frequency

## Discussion and conclusions

Our gene set enrichment analyses were motivated by the goal of making inferences about the mechanism of action of the fictitious drug, assuming that differential methylation could reveal a set of genes associated with a drug that is functionally similar to the fictitious one in question. Upon attending GAW, it became apparent that interaction analysis of the simulated data was not appropriate given the nature of the simulation, and the interaction identified can therefore be considered a false positive. However, reimplementing the same analytic pipeline using the real data produced largely comparable results and identified four pairs of loci between *FTO* and *ALKBH5* with significant interactions. The joint evidence implicating adenosine as the top result from the drug association gene set enrichment analysis and numerous miRNAs involved in metabolic traits from the miRNA target analysis, taken with the assumption that the drug has some unknown epigenetic mechanism, lead us to consider that mRNA or miRNA adenosine methylation, rather than CpG methylation, may be associated with drug response. miRNAs in general are known important regulators of lipid metabolism [8–10]. The top miRNA hit, miR-124a, has evidence for both its role in metabolic traits [11–14] and for its epigenetic regulation in the context of risk of diverse diseases [15–19]. N^6^-Methyladenosine (m^6^A) is a reversible, dynamic posttranscriptional modification that is regulated by miRNAs, and its demethylation has been shown to regulate adipogenesis [20–25]. Recent work demonstrates that RNA conformational changes induced by m^6^A determine substrate specificity for the two RNA demethylases, *FTO* and *ALKBH5* [26–28]. If miRNA adenosine methylation rather than CpG methylation affects the phenotype, although the available data lacks observations on miRNA adenosine methylation, interactions between the two genes that demethylate miRNAs may be biologically relevant and can be assessed with the GOLDN SNP data. Given the evidence for physical interactions between RNA with the m^6^A mark and these demethylases, we were motivated to check for epistasis between SNPs on these genes. Although we did find four pairs of loci with statistically significant interactions, the small sample size means that some SNP-SNP genotypes have few observations, warranting investigation of this interaction in a larger study and further molecular clarification of the distinct and mutual roles of *FTO* and *ALKBH5*. Rather than attempting to explain complex phenotypes solely in terms of single locus main effects, we posit that interaction models better represent the underlying regulatory nature of the genome, and that the joint effect of perturbations to multiple interacting partners can help better explain complex phenotypes. This analysis of epistatic interactions between loci on two genes serves as an illustrative example of how interactions can be significant in the absence of significant main effects, and highlights the need for analyses that integrate multiple sources of data to narrow the search space for plausible interactions.
